# Supplementary material for: Assessment of the performance of nonfouling polymer hydrogels utilizing citizen scientists
Source: PLoS One. 2021 Dec 31;16(12):e0261817. doi: 10.1371/journal.pone.0261817 (PMC8719714; doi:10.1371/journal.pone.0261817)
Supplement: Attachment — Submitted filename: Response to Reviewers.docx [file pone.0261817.s001.docx]

**Journal Requirements:**

1. Please ensure that your manuscript meets PLOS ONE's style requirements, including those for file naming.

To the best of our ability, we have formatted this resubmission to meet PLOS ONE’s style requirements.

2. Acknowledgments Section: Move New Information to the Financial Disclosure:

Please revise the Financial Disclosure statement to the following:

“This research was funded by the National Aeronautics and Space Administration (NASA) as part of the Student Payload Opportunity with Citizen Science (SPOCS) program under Federal Award No 80NSSC17M0021, as a subaward to the University of Idaho. Publication of this article was funded by the University of Idaho - Open Access Publishing Fund. The funders had no role in study design, data collection and analysis, decision to publish, or preparation of the manuscript.”

The Acknowledgments Section has been revised to the following:

“The authors would like to recognize contributions to the project from Dr. Luella Stelck and her students at Russell Elementary School for their contributions to the citizen science and Dr. Stephanie Haag at the University of Idaho for guidance with experimental design.”

3. We note that Figure 1 in your submission contain copyrighted images. All PLOS content is published under the Creative Commons Attribution License (CC BY 4.0), which means that the manuscript, images, and Supporting Information files will be freely available online, and any third party is permitted to access, download, copy, distribute, and use these materials in any way, even commercially, with proper attribution. For more information, see our copyright guidelines: [http://journals.plos.org/plosone/s/licenses-and-copyright](https://urldefense.com/v3/__http:/journals.plos.org/plosone/s/licenses-and-copyright__;!!JYXjzlvb!zdjFwvbJfkQAL77I5Q1eY1V5DV5g-6J4cu4YTc1TR_PSOGXMFo6Vgu_VS7kOyxl69Q$).

We thank the editorial team for checking this – none of the content in Figure 1, including the image on the cover of the citizen science booklet, is copyrighted. We have full, free use of the SPOCS logo. If requested, I can provide a copy of the email from our NASA Program Manager confirming that this image is not copyrighted.

4. Please review your reference list to ensure that it is complete and correct.

We have reviewed the reference list. To the best of our knowledge, it is complete, correct, and none of the cited literature has been retracted. No changes have been made during the revision process.

**Academic Editor**

The study is a well designed and interesting comparison of three common hydrogels as antibacterial surfaces, using data from a large number of students (~100). The research presented is original and to my knowledge not published elsewhere. The experimental methodology is described in good detail, although a breakdown of the individual experimental combinations would improve the clarity of the manuscript (see reviewer 1 below). The conclusions are clear, and the manuscript is well written. I would recommend publication after a few minor alterations suggested by reviewer 1, including a description of how a sample is determined to have the most bacteria, and a break-down of the individual experimental combinations.

Thank you for the positive comments regarding our work. We have addressed both of the concerns raised by the Academic Editor and detailed responses are provided below, within the responses to the comments received from Reviewer #1.

**Reviewer #1**

Bernards and coworkers present an investigation into the antifouling behavior of hydrogels prepared from commercially available monomers. The investigation was performed using Citizen Science and showed that materials made from combinations of TMA/CAA had the highest antifouling behavior. The paper is well written and quite interesting, however, I have some concerns about some of the scientific validity that should be addressed before publication.

We thank the reviewer for their time and effort in reviewing our work and their positive feedback about our study. We have provided detailed responses to each of the specific comments from the reviewer below.

Major concerns:

1) While the methods are clear, the authors have not explained how a sample was deemed to have the most or least bacteria. I assume this is determined by some visual observation, but this needs to be clearly stated either in the main text or in the experimental section. Was the most bacteria determined by the number of distinct colonies, by the sample that showed the single largest colony, by the sample that showed the highest overall cloudiness, etc.?

We thank the reviewer for this suggestion. The samples were deemed to have the most or least bacteria based on the citizen scientist observations of the overall surface coverage of bacteria. The manuscript has been updated in three locations to make this clear as follows:

Page 5, Introduction: “The objective was to rank these three known nonfouling polymers to identify which chemistry demonstrated the best bacteria resistance in a randomized trial based upon the citizen scientist’s observed surface coverage of bacteria.”

Page 6, Results and Discussion: “Following 30 days of repeated bacteria exposure and growth, the citizen scientists were asked to identify the samples that had the greatest and least amounts of bacteria present based on the observed overall surface coverage.”

Page 9, Conclusion: “Samples were ranked as having the least or most bacteria coverage after 30 days of experimentation based on visual observations of surface coverage.”

2) It was clear that the TMA/CAA hydrogel provided the highest antifouling activity overall, however, the data for the individual combinations should also be reported. For instance, in combination 1 (TMA/CAA, TMA/SA, Agar Control), what is the percentage of observations that showed TMA/CAA showed the least bacteria? I am curious to see whether the overall results are skewed by a disproportionately high/low number of observations in one of the combination sets. For instance, did the TMA/CAA show far superior antifouling behavior vs TMA/SA (100% of observations showing TMA/CAA had the least bacteria) but only modest antifouling behavior vs SBMA?

We acknowledge that the reviewer raises an excellent question. We have significantly revised the Results and Discussion section to first provide a breakdown of the results for each combination of experimental variables (Figure 3), followed by the overall summary of the collective results (Figure 4). The revised discussion further strengthens the conclusion that the TMA/CAA chemistry showed the best overall performance.

3) The "multiple" observation in figure 3a is unclear. What happened to this data set when the data was converted to a percentage in figure 3b?

We thank the reviewer for raising this question, and we apologize for this oversight. In the revised presentation of the results in both Figures 3 and 4, all of the “multiple” observations are presented as both a total number of samples and as a percentage of the samples as either “Mix 1”, “Mix 2”, or “Mix 3”. The specific chemistries relating to the “Mix” labels are combination of TMA/CAA and TMA/SA, TMA/CAA and SBMA, and SBMA and Agar, for 1, 2, and 3, respectively.

Minor:

Please define LB (Luria-Bertani) in the experimental procedure section.

We thank the reviewer for catching this omission. We have revised the experimental procedure section to reflect ‘Luria-Bertani” rather than “LB”.

Other notes:

A control sample made from another commercial monomer may have provided a good comparison with the antifouling hydrogels. The antifouling behavior of the TMA/CAA hydrogels may be, in part, due to the presence of residual chemicals during synthesis (e.g. residual unreacted monomers or ethanol). Performing a control experiment with a standard commercial monomer that exhibits little antifouling behavior (e.g. ethylene glycol methyl ether methacrylate) may have more conclusively shown that the enhanced antifouling behavior compared to agar was not due to the synthetic procedure. This will not affect the present study as the comparison was between the 3 hydrogels made via the same procedure.

The reviewer is correct, an additional positive fouling control polymer could have provided additional insight into the performance of the nonfouling hydrogels. At the time of the study design, our focus was to determine the “best” nonfouling polymer over long term exposure to bacteria, for adaptation into the on-going second phase of this work. It is our opinion that the cited literature previously demonstrating the nonfouling properties (references 12, 13, and 15) and biocompatibility (references 21-22) provide evidence that the bacteria adhesion differences are not a result of bacteria death induced by residual chemicals.
